# Physicochemical Characterization of the Loganic Acid–IR, Raman, UV-Vis and Luminescence Spectra Analyzed in Terms of Quantum Chemical DFT Approach

**DOI:** 10.3390/molecules26227027

**Published:** 2021-11-20

**Authors:** Adam Zając, Jacek Michalski, Maciej Ptak, Lucyna Dymińska, Alicja Z. Kucharska, Wiktor Zierkiewicz, Jerzy Hanuza

**Affiliations:** 1 Department of Bioorganic Chemistry, Faculty of Production Engineering, Wroclaw University of Economics and Business, 118–120 Komandorska str., 53-345 Wrocław, Poland; jacek.michalski@ue.wroc.pl (J.M.); lucyna.dyminska@ue.wroc.pl (L.D.); 2 Institute of Low Temperature and Structure Research, 2 Okólna str., 50-422 Wrocław, Poland; m.ptak@int.pan.wroc.pl (M.P.); j.hanuza@int.pan.wroc.pl (J.H.); 3 Department of Fruit, Vegetable and Plant Nutraceutical Technology, Wrocław University of Environmental and Life Sciences, 37 Chełmońskiego str., 51-630 Wrocław, Poland; alicja.kucharska@upwr.edu.pl; 4 Faculty of Chemistry, Wrocław University of Science and Technology, 27 Wybrzeże Wyspiańskiego str., 50-370 Wrocław, Poland; wiktor.zierkiewicz@pwr.edu.pl

**Keywords:** loganic acid, molecular structure, IR, Raman spectra, UV-Vis measurements, luminescence, Stokes shift, quantum chemical DFT calculations

## Abstract

The molecular structure and vibrational spectra of loganic acid (LA) were calculated using B3LYP density functional theory, the 6–311G(2d,2p) basis set, and the GAUSSIAN 03W program. The solid-phase FTIR and FT-Raman spectra of LA were recorded in the 100–4000 cm^−1^ range. The assignment of the observed bands to the respective normal modes was proposed on the basis of the PED approach. The stability of the LA molecule was considered using NBO analysis. The electron absorption and luminescence spectra were measured and discussed in terms of the calculated singlet, triplet, HOMO, and LUMO electron energies. The Stokes shift derived from the optical spectra was 20,915 cm^−1^.

## 1. Introduction

Loganic acid is an iridoid glucoside, an important biologically active component of several fruits such as cornelian cherry, cranberry, bilberry, and honeysuckle berries [1,2,3,4,5,6]. Iridoids comprise a large group of monoterpenoids with six-membered ring skeletons containing oxygen atoms fused to a cyclopentane ring. They can be extracted from the plants both in the state of cyclopentanopyran monoterpenoid glucoside loganic acid and loganin [7]. The conversion of loganic acid into loganin is catalyzed by loganic acid methyltransferase through the transfer of a methyl group from 5-adenosyl-methionine to the carboxylate of loganic acid [7,8,9]. Iridoid glycosides also have numerous biological activities [10] such as antifungal [11] and antimicrobial [12]. They are also used in several medicinal applications [13,14,15,16,17].

It should be noted that several plants can be employed in medicinal applications due to the presence of loganic derivatives in their composition. Phytotherapy enables the support of pharmaceutical treatments at all stages of the disease, proposing new types of remedies [16,18,19,20,21,22,23,24]. Some of them show antidiabetic and antioxidant effects [16,18] due to the significant amounts of iridoid glucosides and loganic acid. Loganic acid also exhibits activity for treating rheumatoid arthritis [19] and inflammation [20]. This iridoid glucoside shows clear neuroprotective effects [21], defends against diet-induced hypertriglyceridemia and atherosclerosis [22], as well as exhibits antiadipogenic effects [23]. Logenic acid reduces cholesterol in the blood and lipid accumulation in the aortic wall [24].

The significant content of loganic acid and its derivatives in several plants had an effect in the new developed techniques of phytopreparation. Several new extraction methods of the bioactive components of the plants were used for isolation of the iridoids. They allowed us to obtain new medicinal remedies with high concentrations of the iridoids and loganic acid [25,26,27,28,29,30,31].

The physicochemical properties and conformation of loganic acid and loganin have rarely been studied and characterized. The ^1^H and ^13^C NMR spectra [32,33] and circular dichroism [34] were measured and analyzed. The XRD structures of loganin and its bromo-derivative were reported in papers [35,36]. The present work reported the results of spectroscopic measurements carried out for iridoid loganic acid. IR, Raman, UV-Vis, and luminescence spectra were analyzed in terms of the quantum chemical DFT approach.

In all the cited papers, the preferable technique used for identification of the iridoids in plants was NMR spectroscopy as a fingerprinting tool for interpretation and quality assessment of natural bioactive products. This method allowed the determination of the amount and structure of the iridoids in the plants. Scores of papers used the 1D and 2D NMR methods for the measurements and interpretation of the ^1^H and ^13^C spectra. Such an approach was applied in several studies in which some selected natural materials were studied [5,14,28,32,34,35,37,38,39,40,41,42,43].

The present work proposed new tools for the identification of loganic acid in plants and their extracts. We reported the results of spectroscopic measurements carried out for iridoid loganic acid. IR, Raman, UV-Vis, and luminescence spectra were analyzed in terms of the quantum chemical DFT approach. The theoretical data were compared with the experimental results obtained from the structural and spectroscopic studies. Such an approach allowed the creation of new tools for the identification of loganic acid, iridoids, and their derivatives present in plants and other natural materials.

## 2. Results and Discussion

### 2.1. Molecular Geometry

**Figure 1:**
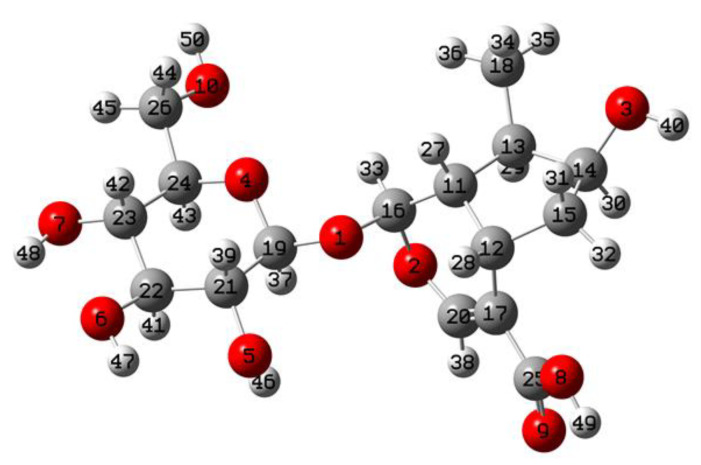
Molecular structure of loganic acid with numbering of atoms.

The optimized molecular structure of loganic acid (LA) with the atoms numbering is shown in Figure 1. The calculated parameters compared to the experimental XRD data reported for loganin [35] are given in Table 1.

### 2.2. Vibrational Spectra

**Figure 2:**
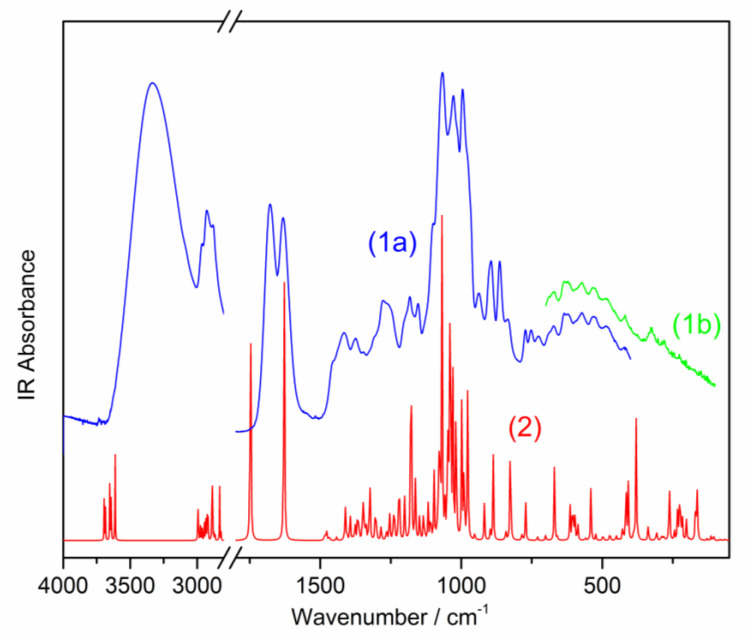
FT-MIR (a) and FT-FIR (b) spectra of the studied loganic acid: (1) experimental spectrum, (2) simulated spectrum.

**Figure 3:**
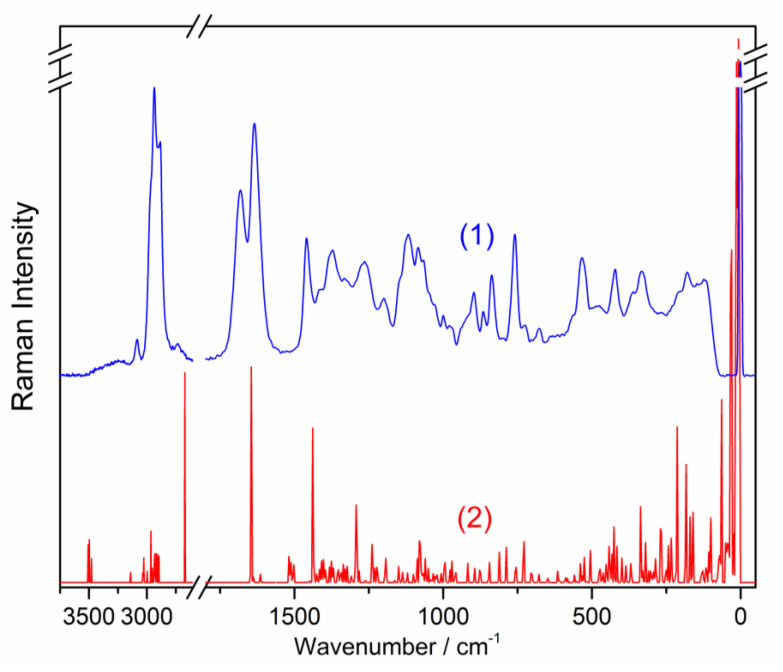
Raman spectra of the studied loganic acid: (1) observed, (2) simulated.

The observed and simulated FTIR and FT-Raman spectra are presented in Figure 2 and Figure 3, respectively. The assignment of the observed bands and calculated wavenumbers to the respective normal modes characterized by the PED procedure is presented in Table 2.

#### 2.2.1. Vibrations of the Cyclopentane[c]pyran System (Φ, φ and Coupled Φφ)

The cyclopentane[c]pyran double-ring (Φφ) system is built of 9 atoms, giving rise to 21 vibrational modes. Ten of them originate from 10 stretching vibrations but the remaining modes correspond to bending and torsion vibrations. Due to the dimeric structure of loganic acid, the number of these modes should double. Therefore, the assignment of the bands presented in Table 2 shows that these modes are characterized by pairs of calculated wavenumbers. They are observed in the range of 800–1650 cm^−1^ in which both IR and Raman spectra contain a very wide spectral pattern that also follows from the existence of one double C=C bond in this system. A total of 21 modes described in Table 2 as vibrations of the cyclopentane[c]pyran double-ring system could be subdivided into the vibrations in which the pyran Φring is involved only, in which the cyclopentane φring participates only and the modes of a mixed nature, and in which the coupled Φφdouble ring system vibrates in the concerted motion. For example, the following bands observed at 1416, 1331, and 1182–1185 cm^−1^ should be assigned to the nearly pure stretching vibrations of the pyran ring, those at about 900–930 and 840 cm^−1^ to the δ(Φ) vibrations, those at 772–780 cm^−1^ to the γ(Φ) vibrations, and those at 80–100 cm^−1^ to the τ(Φ) vibrations. Similarly, the bands observed at about 1408, 1320, 1270, and 1147 cm^−1^ originate from the predominant contribution of the ν(φ) vibrations, those at about 995–999 and 864–865 cm^−1^ to the δ(φ) vibrations, and those at about 250 cm^−1^ to the τ(φ) vibrations. The concerted vibrations in which Φand φrings commonly participate are observed at about 1270–1335, 1180–1270, and 1147 cm^−1^ originating from the stretching motions, at about 968, 894–897, and 835–837 cm^−1^ from in-plane bending δ(Φφ) modes, at about 750–800, 540–600, and 505 cm^−1^ from the out-of-plane bending γ(Φφ) modes, and at about 180–270 and 50–100 cm^−1^ from the torsional τ(Φφ) motions. The appearance and the number of these vibrations confirm that the whole cyclopentane[c]pyran (Φφ) system appears in a sofa conformation, similar to that proposed for loganin [35] and the molecular structure derived from DFT calculations carried out in this paper (Figure 1).

#### 2.2.2. Vibrations of the Pyran Ring

The vibrations of the pyran ring are well defined in the literature [44,45]. The wavenumbers calculated here and their assignment to the respective normal modes agree with these data. The following vibrations have been identified in the present work for this component of loganic acid: ν(θ): 1440–1450, 1372–1374, 1347, 1200–1240, 1150–1153, 1080–1100, and 1028–1029 cm^−1^; δ(θ) at about 979 and 900–930 cm^−1^; γ(θ): 419–421 cm^−1^; and τ(θ) at about 266 and 140–150 cm^−1^. The vibrations of the C-C-H systems of the pyran ring are observed in the following ranges: ν(CH): 3016–3087 cm^−1^; δ(CH): 1100–1480 cm^−1^; ρ(CH): 570–700 cm^−1^; and γ(φ): 450–490 cm^−1^. Other vibrations of the pyran system correspond to the CH_2_ substituent. Its vibrations appear in the ranges: ν_as_(CH_2_): 3050–3080 cm^−1^; ν_s_(CH_2_): 2780–3035 cm^−1^; δ(CH_2_): 1375–1435 cn^−1^; ω(CH_2_): 1120–1365 cm^−1^; τ(CH_2_): 1090–1200 cm^−1^; and ρ(CH_2_): 720–825 cm^−1^.

#### 2.2.3. Carboxylic Group Vibrations

Due to the presence of strong intermolecular hydrogen bonds, carboxylic acids usually exist as dimers. Such an interaction also appears in the case of loganic acid. The recorded IR and Raman spectra exhibit several patterns of bands characteristic for the dimeric arrangement of two carboxylic groups. As an effect of the hydrogen bonds presence, the broad spectral pattern at 2000–3600 cm^−1^ is observed. It results from the overlapping of the bands originating from the vibrations of several O-H groups bonded to the cyclopentane[c]pyran system and pyran rings. The maximum of this band appears at 3334 cm^−1^ (calculated at 3273 cm^−1^). The shape of this broad band can clearly be distinguished from the sharp bands corresponding to the CH, CH_2_, and CH_3_ group vibrations observed in the range of 2700–3100 cm^−1^.

The wavenumber of the bands corresponding to the stretching ν(C=O) vibrations reflects the involvement of the carboxylic CO groups in the HB intermolecular interactions. The vibration wavenumber of this group calculated for the monomeric form of loganic acid is at 1783 cm^−1^, whereas, for the dimer, they are at 1678 and 1671 cm^−1^ for the IR and Raman spectrum, respectively (Table 2). As this band is observed in these spectra at 1677 and 1682 cm^−1^, this confirms the appearance of loganic acid in the dimeric form.

The DFT calculations carried out for the dimeric loganic acid also allowed one to assign other vibrations of its carboxylic group. The C-O stretching and in-plane C-OH bending are expected in the wide range of 1150–1450 cm^−1^ depending on whether the acid is monomeric or dimeric [46]. These vibrations are observed at the following wavenumbers: ν(OH): 3334 cm^−1^; ν(C-COOH): 1200–1202 cm^−1^; γ(OH): 937 and 752–759 cm^−1^; τ(C-COOH): 667, 631–634, and 614–623 cm^−1^; ρ(C-COOH): 532–533; and τ(C-COOH): about 227 cm^−1^.

#### 2.2.4. Vibrations of the C–O–C, i.e., θ-O-ΦBridged Bond

The vibrations of the C–O–C system are described by four normal modes: asymmetric ν_as_(COC) stretching, symmetric ν_s_(COC) stretching, in-plane bending δ(COC), and out-of-plane bending γ(COC) vibrations. The respective wavenumbers observed for loganic acid are located in the following ranges: ν_as_: 1150–1153 cm^−1^; ν_s_: 978–979 and 937–960 cm^−1^; δ: 673–704 cm^−1^; γ: 573–601 cm^−1^; ω: 333 cm^−1^; and τ: 175 and 115 cm^−1^. Apart from these bands, several other modes exhibit participation of the C–O–C vibrations. Such a character appears for the bands observed at the wavenumbers: 1416–1425, 1353–1355, 1340, 1138, 530–533, 433, and 382–386 cm^−1^.

#### 2.2.5. Vibrations of the CH, CH_2_, and CH_3_ Chromophores

Apart from the above-discussed bands, several other vibrations are observed in the analyzed spectra. They correspond to the vibrations of the CH, CH_2_, and CH_3_ substituents of the ring components of loganic acid. They appear in the well-recognized ranges reported in the literature [45]. They are shown in Table 2.

### 2.3. UV-Vis and Luminescence Spectra-Electron States and NBO Analysis

Natural bond orbital analysis is an efficient method reflecting the intra- and inter-molecular interactions in the studied compound, as well as the effect of charge transfer in a molecular system. Table 3 lists the atomic charges calculated for loganic acid.

Analyzing the values of the calculated atomic charges shown in Table 3, the following conclusions can be drawn:

1. The polar character of the C–O–C bridge follows from the positive 0.437 and 0.456 values of the charges on the carbon C_θ_and C_Φ_atoms and the negative value of −0.591 on the oxygen atom.
2. Oxygen atoms inside the θring and Φring exhibit the charges −0.61 and −0.59, respectively, i.e., they have similar character.
3. All the hydroxyl groups appearing in the loganic molecule exhibit similar electron properties—the atomic charges of the oxygen atoms take the values from −0.735 to −0.755.
4. The charges of the carbon atoms differ depending on the place inside the ring and substituent bonded to this atom. The carbon atoms of the pyran θring have positive charge (0.095, 0.107, 0.115, 0.105, and 0.437)—the greatest value corresponds to the atom bonded to the bridging oxygen. Another situation appears in the coupled cyclopentane-pyran Φφsystem. Its carbon atoms change their charge from positive values, 0.456, 0.259, and 0.202, to negative, −0.220, −0.224, −0.245, −0.255, and −0.403. The greatest value appears for the carbon joining the bridging oxygen atom and the smallest value belongs to the C15 atom in the φring.
5. The peculiar situation is observed for the carboxyl group where the C25 atom has charge 0.822, the O9 oxygen atom of the C=O group has charge −0.633, the O8 oxygen atom of the OH group has charge ™0.711, and the H49 hydrogen atom has charge 0.483 (see atomic numbering in Figure 1).

**Figure 4:**
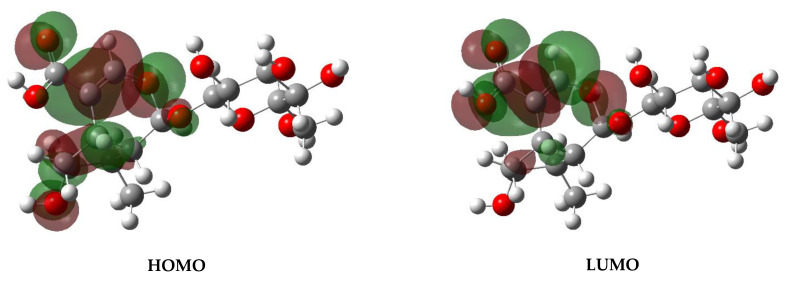
HOMO–LUMO plots of loganic acid.

The above-specified distribution of the atomic charges should strongly influence the electron transitions observed in the UV-Vis and luminescence spectra, and it should especially be reflected in the HOMO–LUMO transitions. The picture of this transition is shown in Figure 4.

**Figure 5:**
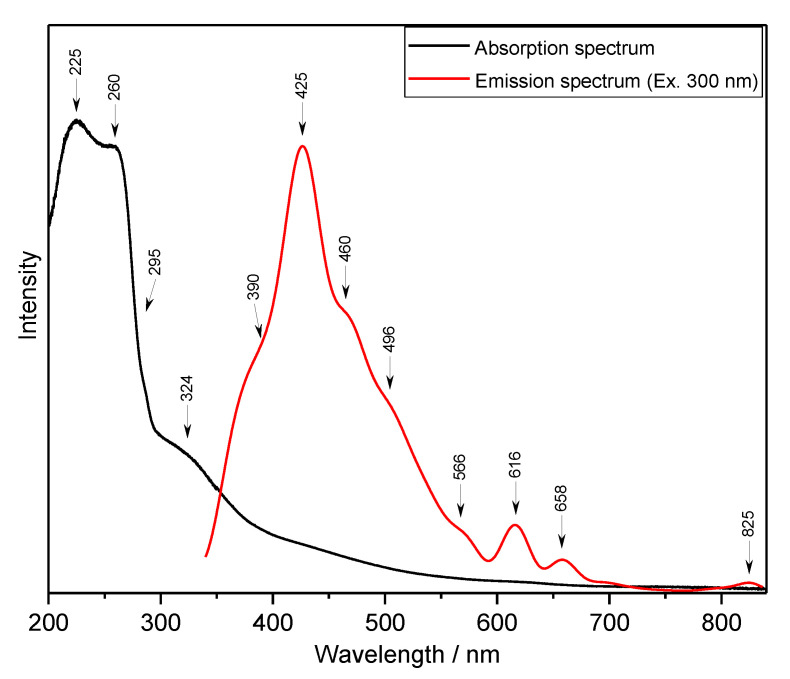
Absorption and emission spectra of loganic acid.

Natural bond analysis is an effective method for studying intra- and inter-molecular bonds, the interaction between bonds, and charge transfer in the molecular system. NBO also informs about interactions between filled donor and empty acceptor orbitals. The results of the theoretical calculation of the HOMO–LUMO energies for the studied compound are shown in Table 4. The calculated values of the chemical hardness, softness, chemical potential, electronegativity, and electrophilicity index of the studied molecule are 2.84, 0.18, −3.78, 3.78, and 2.52, respectively, and are also presented in Table 4. Generally, considering the chemical hardness, a large HOMO–LUMO gap indicates a hard molecule, whereas a small HOMO–LUMO gap represents a soft molecule. The data presented in Table 4 clearly show that the structure of loganic acid is very hard as its HOMO–LUMO energy gap is very large and, simultaneously, its softness is very small. This significant energy gap fits well the large experimental energy transition derived from the measurements of the electron absorption spectra (Figure 5). Besides, the hard structure of loganic acid follows from its dimeric organization and stiffing by hydrogen bonds formed by hydroxyl groups.

The UV-Vis spectrum of loganic acid is shown in Figure 5. The observed bands were assigned to the respective singlet and triplet electron levels calculated for the monomeric form of this compound—they are presented in Table 5.

The wavelengths calculated for the monomeric form of loganic acid can be compared to those obtained for the dimer of this compound. The following fifteen singlet states were derived: characterized by their wavelength and oscillator strength (in parenthesis):

(1) 249.2 (0.2459); (2) 246.8 (0.0027); (3) 245.9 (0.0001); (4) 244.6 (0.0000); (5) 240.8 (0.03165); (6) 239.3 (0.0093); (7) 225.6 (0.1780); (8) 224.8 (0.0023); (9) 223.8 (0.0915); (10) 222.3 (0.0009); (11) 218.1 (0.0004); (12) 217.4 (0.0058); (13) 213.9 (0.0002); (14) 213.5 (0.0024); (15) 213.4 (0.0034). The triplet state energies of the dimer are included in the range of 210–380 nm.

The comparison of the electron energies calculated for the monomer and dimer of loganic acid with the experimental values derived from the UV-Vis spectra allow us to draw the general conclusions of the electronic properties of the studied compound. The calculated energies of the singlet states fit well with those observed in the electronic spectra. The greatest oscillator strengths of these transitions appear for the singlet states located in the range of 200–260 nm and assigned to the bands at 260, 242, 230.5, 212.6, and 210.9 nm. In fact, the greatest intensity is exhibited by the bands observed in the absorption spectra (Figure 5) at 225 and 260 nm. These wavelengths fit well those reported for iridoid glycosides [47]. According to these data, the observed bands correspond to the HOMO→LUMO: *n*→π* transitions. These electron transitions are normally observed in the range of 200–600 nm. The theoretical HOMO→LUMO energy gap for loganic acid is 5.68 eV (45,812 cm^−1^), i.e., at λ = 218 nm. Such a picture suggests that in the HOMO→LUMO excitation, the lone electron pair of the oxygen atom of the hydroxyl group substituted in the cyclopenta[c]pyran system is transferred to the π* antibonding orbitals of the pyran ring of this system.

Such a scheme of these electron transitions is visible in the HOMO–LUMO picture shown in Figure 4. It should be noted that the experimental data fit well the results of the theoretical DFT calculations. The greatest oscillator strengths of the singlet transitions are foreseen in the range of 200–260 nm, which show a good accordance with the absorption bands observed at 225 and 260 nm, i.e., in a very narrow near-UV range. It explains why the color of loganic acid is white. As the range of near-UV is not visible to the human eye and this material scatters all the remaining wavelengths with nearly equal strength, it appears white.

The emission spectrum of loganic acid excited at 330 nm exhibits a strong band at 425 nm and several weak transitions on the slope of this broad band (Figure 5). The comparison of the absorption and emission spectra of loganic acid indicates that the Stokes shift for loganic acid is about 20,915 cm^−1^.

## 3. Materials and Methods

Commercially available loganic acid (PubChem CID: 89640, purity HPLC ≥ 99%) synthesized by the method described in Ref. [48] were taken for measurements.

The attenuated total reflection–Fourier-transform infrared technique and Raman microscopy were used in the measurements of IR and Raman spectra recorded in the ranges of 50–4000 cm^−1^ and 80–4000 cm^−1^. IR spectra were measured using a Nicolet iS50 (Thermo Scientific) spectrometer with a 2 cm^−1^ accuracy. The Raman spectra were collected using a Renishaw InVia Raman spectrometer equipped with a confocal DM 2500 Leica optical microscope—the resolution was 2 cm^−1^.

Room-temperature electron reflectance spectra were measured in the 200–1500 nm spectral range using a Cary-Varian 5E UV-VIS-near-IR spectrophotometer. The spectra were recorded with Praying Mantis diffuse reflectance accessories. In these measurements, the baseline was first recorded for Al_2_O_3_ powder, and this line was then subtracted from that obtained for particular powder sample spectra. The absorption spectra of the ground complexes in silicon paste were recorded.

Emission spectra and luminescence decay curves were recorded with a grating spectrograph (Princeton Instr. Model Acton 2500i) coupled to a streak camera (Hamamatsu Model C5680) operating in the 200–1100 nm spectral range with a temporal resolution of 20–100 ps. The luminescent properties of the complexes were investigated at room temperature.

The molecular structure of loganic acid was optimized at the DFT (Density Functional Theory) level using the Becker–Lee–Yang–Parr correlation functional and the 6–311G(2d,2p) basis set using the GAUSSIAN 03W program. The results of the DFT calculations were used in interpretation and analysis of the experimental spectroscopic studies. The mean square deviation between the experimental and calculated unscaled wavenumbers was nearly 25 cm^−1^ for the IR and Raman spectra. The scaling of the calculated wavenumbers improved this result to 5 cm^−1^ for the IR and 24 for the Raman spectra. A scaling factor of 0.96 was used for the range of 3700–2500 cm^−1^ and 0.98 for the range of 2499–50 cm^−1^ of the spectra.

## 4. Conclusions

Loganic acid appears in the solid state in a dimeric structure forming white small-crystalline powder. Its structural parameters were calculated using the DFT B3LYP/6–311G(2d,2p) approach. It was shown that these geometrical data fit well the structure of loganin determined in the XRD experiment.

IR and Raman spectra proved that loganic acid occurs in a dimeric form due to the existence of intermolecular C^OH^^⋅⋅⋅⋅O^_O__⋅⋅⋅HO_C hydrogen bonds between the adjacent molecules. It was confirmed by the DFT calculations carried out for both its monomeric and dimeric forms. The calculations carried out for these systems were the basis for the assignment of all the observed IR and Raman bands to the respective molecular vibrations.

The most characteristic vibrations observed in the vibrational spectra include those related to the C–O–C (i.e., θ-O-Φ) bridge bond. They were observed at the following wavenumbers: ν_as_: 1150–1153 cm^−1^; ν_s_: 937–979 cm^−1^; δ: 673–704 cm^−1^, γ: 573–601 cm^−1^, ω: 333 cm^−1^; and τ: 115–175 cm^−1^.

The atomic charges calculated using DFT proved that the C–O–C bridge has a strong polar character due to clear positive charges on the carbon atoms and the negative charge of the oxygen atom.

The electron properties of loganic acid are dominated by the electronic structure of the double cyclopentane-pyran system. In the HOMO→LUMO excitation, the lone electron pairs of the oxygen atoms from the hydroxyl groups are transferred to π* antibonding orbitals. The *n*(O)→π* transition corresponds to the strong and broad band observed both in the UV-Vis and luminescence spectra of this compound.

The composition and electron structure of loganic acid suggest that this compound can be used as a complexing ligand binding the d-electron and f-electron metal ions. The binding of such ions can be fulfilled by carboxyl and OH groups of the ligands. The energies of the singlet and triplet levels determined for loganic acid are convenient for its excitation and energy transfer to a metal ion.

## Figures and Tables

**Table 1:** Optimized geometrical parameters of loganic acid obtained by DFT calculations compared with the experimental structural data reported for loganin [35].

Bond Distances	Calc.	Exp.	Bond Angles	Calc.	Exp.
O1—C16	1.442	1.426	C11—O1—C19	115.5	114.7
O2—C16	1.475	1.432	O1—C16—O2	108.7	109.6
O2—C20	1.374	1.357	C16—O2—C20	116.6	115.0
C20—C17	1.354	1.333	O2—C20—C17	125.1	124.4
C17—C12	1.502	1.510	C20—C17—C12	122.0	123.2
C12—C11	1.561	1.549	C17—C12—C11	110.7	110.7
C11—C16	1.515	1.509	C17—C12—C15	113.9	112.3
C12—C15	1.551	1.551	C12—C11—C16	113.2	113.0
C15—C14	1.139	1.522	C11—C16—O2	111.6	113.1
C14—C13	1.562	1.522	O1—C19—O4	107.7	107.5
C13—C11	1.566	1.535	C19—O4—C24	1132	111.7
C17—C25	1.466	1.482	O4—C24—C23	107.5	108.8
C25—O9	1.243	1.197	C24—C23—C22	110.0	110.7
C25—O8	1.389	1.341	C23—C22—C21	111.3	111.1
C14—O3	1.457	1.439	C22—C21—C19	109.1	108.4
C13—C18	1.537	1.528	C12—C15—C14	104.8	106.3
O1—C19	1.432	1.396	C15—C14—C13	105.8	103.9
O4—C19	1.448	1.433	C12—C11—C13	106.8	104.4
O4—C24	1.473	1.422	C16—C11—C13	112.6	114.2
C23—C24	1.534	1.530	C20—C17—C25	115.5	120.0
C23—C22	1.526	1.529	C17—C25—O9	126.7	123.5
C22—C21	1.522	1.515	C17—C25—O8	113.0	113.4
C21—C19	1.523	1.511	C15—C14—O3	113.1	111.9
C24—C26	1.512	1.507	C14—C13—C18	114.1	115.5
C26—O10	1.453	1.418	O4—C24—C26	107.8	107.0
C23—O7	1.447	1.415	C24—C23—O7	108.2	112.1
C22—O6	1.450	1.414	C23—C22—O6	106.6	111.1
C21—O5	1.449	1.419	C22—C21—O5	106.3	108.7
**Torsion angles**			**Calc.**	**Exp.**	
C20—C17—C12—C15			−122.4	−117.3	
O2—C16—C11—C13			68.0	67.5	
C16—O1—C19—O4			−67.2	−87.5	
C16—O1—C19—C21			174.9	153.2	
C14—C13—C11—C16			−138.5	−165.4	

**Table 2:** Calculated and experimental vibrational wavenumbers with assignments for loganic acid vibrations.

No.	Calculated			Experimental		Assignment for Monomer
	dimer		monomer	IR	RS	
1	3582	3582	3695(12,2)			ν(OH)-θ(99)
2	3579	3579	3684(11,2)			ν(OH)-φ(98)
3	3571	3571	3653(17,1)			ν(OH)-φ(99)
4	3570	3570	3650(6,0)			ν(OH)-θ(98)
5	3551	3551	3642(17,1)		3400 sh	ν(OH⋅⋅⋅O)-θ(99)
6	3208	3208	3613(28,2)	3334 vs,b		ν(OH⋅⋅⋅O)-Φ(98) (A group)
7	3098	3098	3081(0,2)			ν(CH)-Φ(99)
8	3091	3091	2995(7,2)	3086 sh	3083 w	ν(CH)-Φ(99)
9	3089	3089	2993(7,2)			νas(CH_3_)-Φ(99)
10	3061	3061	2998(3,2)			ν_as_(CH_2_)-φ(89)
11	3026	3026	2974(10,1)			νas(CH_3_)-φ(97)
12	3025	3025	2964(5,1)			ν(CH)-Φ(94)
13	3014, 3008	3014, 3008	2946(10,3)			ν_as_(CH_2_)-θ(93)
14	2999	2999	2936(2,1)			ν(CH)-Φ(92)
15	2997	2997	2934(10,2)			ν(CH)-θ(74)
16	2992	2992	2925(6,2)			νas(CH_2_)-φ(82)
17	2987	2987	2922(4,3)			ν(CH)-θ(85)
18	2980	2980	2917(7,3)			ν(CH)-φ(75)
19	2971	2971	2889(28,1)			νs(CH_3_)-φ(93)
20	2967	2967	2885(8,1)	2963 s	**2963 sh**	ν(CH)-θ(98)
21	2958	2958	2876(2,3)			ν(CH)-θ(90)
22	2956	2956	2868(2,3)	2928 s	2936 vs	νs(CH_2_)-θ(91)
23	2867		2833(16,3)	2882 s	2884 s	ν(CH)-θ(96)
24		2726	2822(2,0)			νs(CH_2_)-φ(89)
25	1644	1638	1747(92,2)	1677 s	1682 s	ν(C=O)-Φ(A group) (69)
26	1615	1605	1623(100,8)	1632 s	1634 vs	ν(Φ) (45) + ν(C=O) (A group) (17)
27	1519	1519	1486(2,1)	**1517 vw**	**1526 vw**	δ_as_(CH_2_)-θ(75) + δ(COH)-θ(11)
28	1517	1517	1481(1,1)			δ_as_(CH_2_)-φ(77)
29	1511	1511	1477(2,1)	1490 sh		δ_as_(CH_3_)-φ(87)
30	1503	1503	1468(2,1)	1481 sh		δ_as_(CH_3_)-φ(90)
31	1460		1442(1,0)	1458 w	1459 m	δ(CH_2_OH)-θ(72)
32	1449	1449	1415(2,1)	**1454 sh**		δ(COH)-θ(42) + δ(CCH)-θ(31)
33	1439		1404(9,0)			δ_s_(CH_3_)-φ(56) + δ(CCH)-φ(24)
34	1435	1434	1405(1,0)			δ(COH)-θ(53) + δ(CCH)-θ(34)
35	1425	1425	1404(1,1)			ν(θ) (49) + ν(COC) (25)
36	1416	1416	1396(1,1)	1416 w	1416 sh	ν(θ) (43) + ν(COC) (30)
37	1410	1410	1394(7,0)	1412 sh	1408 sh	ν(φ) (59)
38	1403	1403	1385(1,0)			ν(φ) (49) + δ(COH)-φ(34)
39	1403	1402	1377(6,0)	1405 sh		ν(θ) (58) + δ(CCH)-θ(24)
40	1397	1397	1375(0,1)			ν(Φ) (93)
41	1382	1382	1368(5,1)		1384 sh	ν(φ) (84)
42	1376	1376	1363(9,1)	1378 sh		ν(φ) (78)
43	1371	1371	1349(9,0)	1374 w	1372 m	ν(φ) (38) + δ(CCH)-φ(29)
44	1369	1369	1347(13,0)	1363 sh		ν(θ) (54) + δ(CCH)-θ(27)
45	1355	1355	1341(2,0)			ν(COC) (27) + δ(CCH)-Φ(25)
46	1353	1353	1337(4,1)			ν(COC) (29) + ν(Φ) (25)
47	1351	1351	1329(3,1)			ν(Φ) (72)
48	1345	1345	1325(5,0)	1347 w		ν(θ) (50) + δ(COH)-θ(25)
49	1337	1337	1324(16,0)	1340 sh		ν(θ) (55) + ν(COC) (32)
50	1330	1330	1304(14,0)		1331 w	ν(φ) (44) + δ(CCH)-φ(23)
51	1323	1323	1299(2,0)			ν(θ) (60) + δ(COH)-θ(21)
52	1308	1308	1284(6,0)	1310 sh		ν(φ) (41) + δ(CCH)-φ(35)
53	1301	1295	1267(1,1)			ν(Φ) (47) + δ(CCH)-Φ(22)
54	1295	1294	1264(2,0)		1295 sh	ν(φ) (35) + δ(CCH)-φ(24)
55	1293	1292	1254(11,0)			ν(C-CH_3_)-θ(54) + δ(COH)-θ((19)
56	1283	1283	1241(5,0)	1276 m	1276 sh	δ(CH_2_)-θ(64) + δ(COH)-θ(34)
57	1261	1261	1238(12,1)	1260 sh	1264 w	δ(COH)-θ(55)
58	1239	1239	1223(6,0)		1254 sh	ν(φ)-(49) + δ(CCH)-φ(29)
59	1238	1238	1221(8,0)			δ(COH)-φ(44) + δ(CCH)-φ(35)
60	1230	1230	1219(12,1)			δ(CCH)-θ(71) + ν(C–C)-θ(24)
61	1222	1222	1201(15,0)			ν(φ) (35) + δ(CCH)–Φ(34) + ν(Φ) (29)
62	1220	1239	1180(31,0)			ν(θ) (53) + δ(CCH)-θ(27) + δ(COH)-θ(10)
63	1196	1194	1177(33,1)	1202 sh	1200 w	ν(φ) (47) + δ(C-COOH)-Φ(38)
64	1191	1191	1163(22,0)	1182 m	1185 sh	δ(C-COOH)-Φ(44) + ν(Φ) (41)
65	1163	1162	1148(9,1)			ν(Φ) (41) + δ(CCH)-Φ(36)
66	1149	1148	1134(7,2)	1153 m	1150 sh	ν_as_(C–O–C) (53) + ν(C-COH)-θ(41)
67	1138	1138	1130 (4,0)			ν(C-CH_3_)-φ(76) + ν(C-C)-θ(22)
68	1136	1136	1117(11,0)			ν(θ) (96)
69	1121	1121	1108(10,1)	1121 sh	1124 sh	ν(C-CH_3_)-φ(65) + ν(C-OH)-φ(30)
70	1101	1101	1097(20,1)		1117 m	ν(C-OH) θ(51) + ν(θ) (45)
71	1088	1088	1091(1,1)	1099 sh		ν(θ) (55) + δ(COH)-θ(42)
72	1080	1078	1083(10,1)		1083 m	ν(θ) (74) + δ(COH)-θ(22)
73	1077	1075	1077(51,3)			ν(θ) (52) + δ(COH)-θ(46)
74	1072	1069	1068(92,0)		1067 w	δ(COH)-θ(54) + ν(C–C)-θ(42)
75	1066	1064	1058(9,1)	1066 vs		ν(φ) (71) + δ(CCH)-φ(26)
76	1059	1058	1049(37,1)			ν(θ) (53) + ν(COH)-θ(43)
77	1051	1051	1046(20,1)		1047 sh	ν(C-OH)-φ(48) + ν(C-CH_3_)-φ(41)
78	1043	1042	1040(56,0)			ν(φ) (54) + δ(CCH)-φ(44)
79	1035	1034	1039(54,1)			ν(θ) (94)
80	1026	1026	1029(50,0)	1028 vs	1029 sh	ν(φ) (46) + ν(Φ) (45)
81	1021	1019	1018(49,0)			ν(C-OH)-φ(51) + δ(C-CH_3_)-φ(44)
82	1015		999(39,0)	**1016 sh**		δ(φ) (96)
83	1006	1006	990(28,0)			δ(θ) (96)
84	997	997	979(6,0)	995 vs	999 w	δ(Φ) (97)
85	994	993	978(11,1)			δ(θ) (55) + ν_s_(C–O–C) (43)
86	978	977	977(36,1)	**978 sh**	979 vw	δ(θ) (66) + ν_s_(C–O–C) (32)
87	971	971	971(11.1)	**967 sh**	968 sh	δ(θ) (52) + ν(C-OH) (41)
88	959	958	952(2,0)	937 w	937 sh	ν_s_(C–O–C) (52) + δ(Φ + φ) (46)
89	917	916	918(11,0)	901 sh		γ(C-CH_3_)-φ(74) + δ(φ) (23)
90	895	894	897(3,1)	894 m	897 w	δ(θ) (55) + δ(C-CH_3_)-θ(41)
91	876	876	886(25,1)	864 m	865 w	δ(φ) (94)
92	846	846	843(4,2)	835 w	837 w	γ(Φ) (95)
93	812	811	826(36,1)		806 vw	γ(Φ-φ) (88)
94	789	789	784(2,3)	772 w	796 vw	γ(Φ-COOH) (68) + γ(Φ) (31)
95	757	756	772(12,1)	752 w	759 m	γ(Φ-COOH) (48) + γ(Φ) (46)
96	741	729	730(1,5)	726 w	725 vw	γ(Φ-COOH) (61) + γ(Φ) (33)
97	704	701	700(2,1)		704 vw	δ(C–O–C) (49) + τ(Φ-COOH) (43)
98	682	679	669(22,2)	673 w	678 vw	δ(C–O–C) (78) + ρ(Φ-COOH) (21)
99	650	650	660(1,0)	667 sh		τ(Φ-COOH) (86) + γ(φ) (13)
100	615	615	614(10,0)	634 w	631 vw	τ(θ-CH_2_OH) (48) + τ(φ-COOH) (43)
101	609	609	606(11,1)	623 w	614 vw	τ(Φ-COOH) (61) + γ(φ) (33)
102			603(5,0)		601 vw	γ(C–O–C) (51) + τ(Φ-COOH) (38)
103	590	590	598(8,2)	593 vw		γ(θ) (47) + γ(θ-OH) (44)
104	583	583	587(6,1)		587 sh	γ(φ) (58) + τ(φ-CH_3_) (43)
105	564	560	560 (1,1)	573 w		γ(θ-O-Φ) (93)
106	551	538	540(16,1)	538 sh		γ(Φ) (89)
107	532	526	523(2,2)	532 w 530 w	533 m	τ(Φ-COOH) (64) + γ(Φ-C–O–C) (32)
108	517	505	497(2,1)	522 sh 505 w		τ(θ-OH) (39) + τ(θ-CH_2_OH) (37) + γ(θ) (20)
109	476	471	472(3,1)	487 w	485 w	γ(φ) (56) + τ(θ-OH) (37)
110	465	462	451(2,2)		475 vw	τ(θ-OH) (61) + γ(θ) (37)
111	452	448	442(1,1)	455 vw		τ(θ-OH) (54) + γ(θ) (36)
112	441	441	428(5,2)			τ(θ-OH) (62) + τ(θ) (31)
113	433	433	417(20,3)			τ(θ) (47) + γ(θ-O-Φ) (43)
114	425	424	409(17,1)		421 w	τ(φ-OH) (34) + γ(Φ) (32) + τ(θ) (31)
115	417	416	389(4,1)	419 vw		γ(θ) (51) + τ(θ-OH) (43)
116	400	399	380(35,4)			τ(φ-OH)(31) + τ(φ-CH_3_) (30) + τ(θ-CH_2_OH) (29)
117	386	382	371(1,1)			τ(φ-CH_3_) (33) + τ(θ-OH) (32) + τ(θ-O-Φ) (32)
118	370	368	337(6,5)		362 sh	γ(θ) (64) + τ(θ-CH_2_OH) (32)
119	336	335	312(1,2)		333 w	ω(θ-O-Φφ) (94)
120	329	319	307(2,1)	328 vw		τ(HO-θ-OH) (48) + τ(HO-φ/Φ) (45)
121	310	302	291(1,3)			τ(HO-φ-CH_3_) (94)
122	298	292	287(1,1)			τ(HO-φ-CH_3_) (68) + ρ(θ-OH) (37)
123	287	287	281(2,1)			ρ(φ-CH_3_) (38) + ρ(θ-OH) ((29) + ρ(φ-OH) (29)
124	284	283	263(21,0)			τ(φ-OH) (95)
125	270	268	244(3,1)		266 w	τ(HO-θ-OH) (53) + τ(θ) (41)
126	263	251	233(8,2)			τ(θ-CH_2_OH) (57) + τ(HO-θ-OH) (41)
127	244	244	228(5,2)			τ(φ) (47) + τ(φ-COOH) (45)
128	234	233	224(18,3)			τ(φ-CH_3_) (96)
129	230	230	217(10,3)	227 vw		τ(θ-CH_2_OH) (53) + τ(HO-θ-OH) (46)
130	221	218	202(7,3)			τ(φ-COOH) (66) + τ(φ) (31)
131	215	214	191(0,2)		204 sh	τ(θ-CH_2_OH) (93)
132	184	184	171(7,3)			τ(Φ + φ) (98)
133	171	170	165(23,14)		175 w	τ(θ-O-Φ) (96)
134	161	160	160(2,6)	150 vw	146 w	τ(θ) (56) + τ(θ-CH_2_OH) (29) + τ(θ-O-Φ) (11)
135	132, 126	130	128(1,1)		125 w	τ(θ-CH_2_OH) (49) + τ(HO-θ-OH) (43)
136	115	116	115(1,4)		115 sh	τ(HO-θ-OH) (34) + τ(θ-CH_2_OH) (36)+ τ(θ-O-Φ) (23)
137	107	101	106(2,1)			τ(Φ-φ) (42) + τ(C-COOH)-Φ(33)
138	102, 97	89	103(2.2)			τ(θ-COH) (49) + τ(θ-CH_2_) (45)
139	78	80	82(0,3)			τ(Φ-COOH) (58) + τ(Φ) (44)
140	73	72	74(0,5)			τ(Φ) (54) + ρ(C-COOH)-Φ(33)
141	65	64	65(1,21)			τ(θ) (48) + ρ(COH)-θ(40)
142	39	38	39(0,32)			τ(θ-Φ) (57) + ρ(COH)-φ(36)
143	33	30	32(0,25)			τ(θ-Φφ(70) + τ(COH)-φ(19)
144	24	22	21(0,100)			τ(θ-Φφ) (79)

Abbreviations used: s—strong; m—medium; w—weak; v—very; sh—shoulder; ν—in-plane stretching vibrations (s—symmetric; as—asymmetric); δ—in-plane bending vibrations; γ—out-of-plane bending vibrations; τ—torsional; ρ—rocking; ω—wagging vibrations. θ—carbohydrate ring; Φ—pyran ring of the cyclopenta[c]pyran (iridoid) system; φ—cyclopentane ring of the cyclopenta[c]pyran system; θ-O-Φ and C–O–C—glycosidic bridge bond; COO—carboxyl group. Used scaling factor: for monomer fsc = 0.96 (2500–3700 cm^−1^), fsc = 0.98 (0–2500 cm^−1^); for dimer fsc = 0.98 (0–3700 cm^−1^).

**Table 3:** Atomic charges of loganic acid derived from the NBO analysis.

Atomic Numbering	Mulliken Atomic Charges	NBO Atomic Charge	Atomic Numbering	Mulliken Atomic Charges	NBO Atomic Charge
O1	−0.548	−0.591	C26	0.251	−0.015
O2	−0.470	−0.549	H27	0.037	0.223
O3	−0.503	−0.749	H28	0.058	0.225
O4	−0.548	−0.610	H29	0.049	0.200
O5	−0.522	−0.736	H30	0.035	0.161
O6	−0.544	−0.755	H31	0.049	0.202
O7	−0.545	−0.748	H32	0.047	0.211
O8	−0.430	−0.711	H33	0.063	0.191
O9	−0.430	−0.633	H34	0.061	0.197
O10	−0.500	−0.735	H35	0.063	0.208
C11	−0.083	−0.245	H36	0.057	0.192
C12	−0.085	−0224	H37	0.002	0.144
C13	−0.127	−0.220	H38	0.073	0.201
C14	0.278	0.166	H39	0.057	0.196
C15	−0.067	−0.403	H40	0.235	0.455
C16	0.591	0.456	H41	0.022	0.157
C 17	−0.201	−0.255	H42	0.036	0.169
C18	−0.119	−0.563	H43	0.041	0.179
C19	0.473	0.437	H44	0.033	0.160
C20	0.275	0.259	H45	0.042	0.176
C21	0.246	0.095	H46	0.247	0.454
C22	0.246	0.107	H47	0.256	0.470
C23	0.275	0.115	H48	0.263	0.470
C24	0.180	0.105	H49	0.249	0.483
C25	0.594	0.822	H50	0.243	0.455

**Table 4:** The calculated HOMO–LUMO energy gaps and quantum chemical properties of title compound at DFT/B3LYP.

No	Molecular Orbitals	Energy	Energy Gap	Ionization Potential I	Electron Affinity A	Global Hardness η	Chemical Potential μ	Electro-Negativity χ	Global Softness σ	Global Electrophilicity ω
		[eV]	[eV]	[eV]	[eV]	[eV]	[eV]	[eV]	[eV]	[eV]
1.	H	−6.62	5.68	6.62	0.94	2.84	−3.78	3.78	0.18	2.52
	L	−0.94								
2.	H − 1	−7.30	7.74	7.30	0.44	3.87	−3.43	3.43	0.13	1.53
	L + 1	0.44								
3.	H − 2	−7.42	8.15	7.42	0.73	4.08	−3.35	3.35	0.12	1.38
	L + 2	0.73								

H: HOMO, L: LUMO, I = −E_HOMO_, A = −E_LUMO_.

**Table 5:** Calculated singlet and triplet electron levels of loganic acid in the monomeric form.

Electron Levels	eV	nm	cm^−1^	Oscillator Strength
**Singlets**				
S1	5.1236	241.99	41,324	0.0073
S2	5.3784	230.52	43,380	0.2038
S3	5.8312	212.62	47,032	0.0182
S4	5.8778	210.94	47,407	0.0516
S5	6.2700	197.74	50,571	0.0001
S6	6.3440	195.44	51,167	0.0008
S7	6.4366	192.62	51,916	0.0041
S8	6.5325	189.80	52,687	0.0020
S9	6.5971	187.94	53,208	0.0020
S10	6.6393	186.74	53,550	0.0004
**Triplets**				
T1	3.4084	363.76	27,491	0.0000
T2	4.7521	260.90	38,329	0.0000
T3	5.5947	221.61	45,124	0.0000
T4	5.7579	215.33	46,440	0.0000
T5	5.8279	212.74	47,006	0.0000
T6	6.2618	198.00	50,505	0.0000
T7	6.3102	196.48	50,896	0.0000
T8	6.3757	194.46	51,424	0.0000
T9	6.4022	193.66	51,637	0.0000
T10	6.4411	192.49	51,951	0.0000

## Data Availability

The data presented in this study are available on request from the corresponding author.

## Sample Availability

Samples of the compounds are not available from the authors.
